# Implementation and Application of Telemedicine in China: Cross-Sectional Study

**DOI:** 10.2196/18426

**Published:** 2020-10-23

**Authors:** Fangfang Cui, Qianqian Ma, Xianying He, Yunkai Zhai, Jie Zhao, Baozhan Chen, Dongxu Sun, Jinming Shi, Mingbo Cao, Zhenbo Wang

**Affiliations:** 1 National Engineering Laboratory for Internet Medical Systems and Applications The First Affiliated Hospital of Zhengzhou University Zhengzhou, Henan China; 2 National Telemedicine Center of China Zhengzhou, Henan China; 3 School of Management Engineering Zhengzhou University Zhengzhou, Henan China

**Keywords:** telemedicine, Chinese hospital, implementation, application, influencing factors

## Abstract

**Background:**

Telemedicine has been used widely in China and has benefited a large number of patients, but little is known about the overall development of telemedicine.

**Objective:**

The aim of this study was to perform a national survey to identify the overall implementation and application of telemedicine in Chinese tertiary hospitals and provide a scientific basis for the successful expansion of telemedicine in the future.

**Methods:**

The method of probability proportionate to size sampling was adopted to collect data from 161 tertiary hospitals in 29 provinces, autonomous regions, and municipalities. Charts and statistical tests were applied to compare the development of telemedicine, including management, network, data storage, software and hardware equipment, and application of telemedicine. Ordinal logistic regression was used to analyze the relationship between these factors and telemedicine service effect.

**Results:**

Approximately 93.8% (151/161) of the tertiary hospitals carried out telemedicine services in business-to-business mode. The most widely used type of telemedicine network was the virtual private network with a usage rate of 55.3% (89/161). Only a few tertiary hospitals did not establish data security and cybersecurity measures. Of the 161 hospitals that took part in the survey, 100 (62.1%) conducted remote videoconferencing supported by hardware instead of software. The top 5 telemedicine services implemented in the hospitals were teleconsultation, remote education, telediagnosis of medical images, tele-electrocardiography, and telepathology, with coverage rates of 86.3% (139/161), 57.1% (92/161), 49.7% (80/161), 37.9% (61/161), and 33.5% (54/161), respectively. The average annual service volume of teleconsultation reached 714 cases per hospital. Teleconsultation and telediagnosis were the core charging services. Multivariate analysis indicated that the adoption of direct-to-consumer mode (*P*=.003), support from scientific research funds (*P*=.01), charging for services (*P*<.001), number of medical professionals (*P*=.04), network type (*P*=.02), sharing data with other hospitals (*P*=.04), and expertise level (*P*=.03) were related to the effect of teleconsultation. Direct-to-consumer mode (*P*=.01), research funding (*P*=.01), charging for services (*P*=.01), establishment of professional management departments (*P*=.04), and 15 or more instances of remote education every month (*P*=.01) were found to significantly influence the effect of remote education.

**Conclusions:**

A variety of telemedicine services have been implemented in tertiary hospitals in China with a promising prospect, but the sustainability and further standardization of telemedicine in China are still far from accomplished.

## Introduction

Telemedicine refers to the remote delivery of health care services with the use of modern communication technology, electronic technology, and multimedia computer technology to realize the remote collection, transmission, processing, storage, and inquiry of medical information and to further provide the examination, surveillance and diagnosis of disease, remote education, and information management [1,2]. The common types of telemedicine services include teleconsultation, telediagnosis, and remote surgery teaching [3-5]. With the rapid development of telemedicine equipment and information communication technology, telemedicine has developed rapidly and is used widely around the world as a new mode of medical service [6,7]. In particular, in China, telemedicine has been used as a crucial method by the government to address the inequality of medical resources between urban and rural areas.

Medical care is an important issue related to the national economy and people's livelihood in China. However, there is a serious imbalance of distribution in medical resources, and patients in rural and remote areas do not have easy access to high-quality medical services [8]. In 2018, there were 1047 tertiary hospitals in 11 provinces in the east of China and 1216 tertiary hospitals in 21 provinces in the central and western regions [9]. High-quality medical resources are concentrated in the economically developed eastern region of China, while the central and western regions have a serious lack of health resources [10]. A health report showed there were 10.91 health care technicians per 1000 people in China’s urban areas in 2018, while there were only 4.63 health care technicians per 1000 people in rural areas. Moreover, the number of beds per 1000 people in urban medical institutions was 8.70, while it was only 4.56 in rural areas, and there were less than 2 medical beds in township health centers per 1000 rural people [9]. The imbalanced distribution of medical resources in urban and rural areas greatly increases the difficulty of medical treatment in rural areas and reduces the efficiency of treatment [11,12]. Medical experts from large hospitals in developed cities can provide telediagnosis for patients in rural and remote areas through telemedicine, which may shorten the spatial distance between doctors and patients and enable patients to be treated locally by off-site medical experts. Numerous studies have shown that telemedicine can effectively reduce expenses and save time for medical treatment [13-16]. Therefore, telemedicine has been used as an important means for medical reform, and many policies have been issued to encourage the development of telemedicine in China.

The development of telemedicine in China began in the 1980s. In 1986, the Guangzhou Ocean Shipping Company conducted a cross-sea consultation for the emergency patients on the ocean-going freighter through telegraph, which was considered to be the earliest telemedicine activity in China. In 1997, the Jinwei Medical Network in China was officially opened to provide remote, off-site, real-time, and dynamic live television consultations for patients with severe illness [17]. Subsequently, the medical institutions at all levels in China began to explore and develop telemedicine [18,19]. After years of efforts, the development of telemedicine entered its golden age. In 2017, 22 provinces in China established telemedicine platforms covering 13,000 medical institutions, providing teleconsultation, telediagnosis, and remote medical education [20].

Many scholars have carried out research on telemedicine, as telemedicine has been implemented in full swing in China. Several studies have analyzed the application of telemedicine in the treatment of different diseases such as diabetes and burns [21-23]. Further, Cai et al [19] investigated the experience of doctors and patients in the implementation of telemedicine in the Gansu Province, China. He et al [24] analyzed the patient satisfaction and compliance with telemedicine implementation in rural areas in the Guangdong Province, China. However, most of the researches focused on the application of telemedicine in certain diseases or were limited to a certain region (such as a province) or small aspects such as patient satisfaction. Research on the overall development of telemedicine in China from a national and comprehensive perspective has not been elucidated. In order to explore the characteristics of telemedicine in a multidimensional and in-depth manner, this study investigated the development of telemedicine in 161 tertiary hospitals in 29 provinces, autonomous regions, and municipalities, and conducted an overall analysis of the implementation, applications, and key factors of telemedicine in China in 2017. This study is the first to summarize the development of telemedicine in China with comprehensive information rather than a small aspect, including human resources, network construction, hardware and software facilities, and so on, thereby showing how the telemedicine was built and operated in China. Besides, this study carried out a nationwide survey, not limited to a certain area, which is unprecedented. Furthermore, the overview of implementation and application of telemedicine would be valuable for formulating policies and improving existing deficiencies.

## Methods

### Study Design and Participants

**Figure 1:**
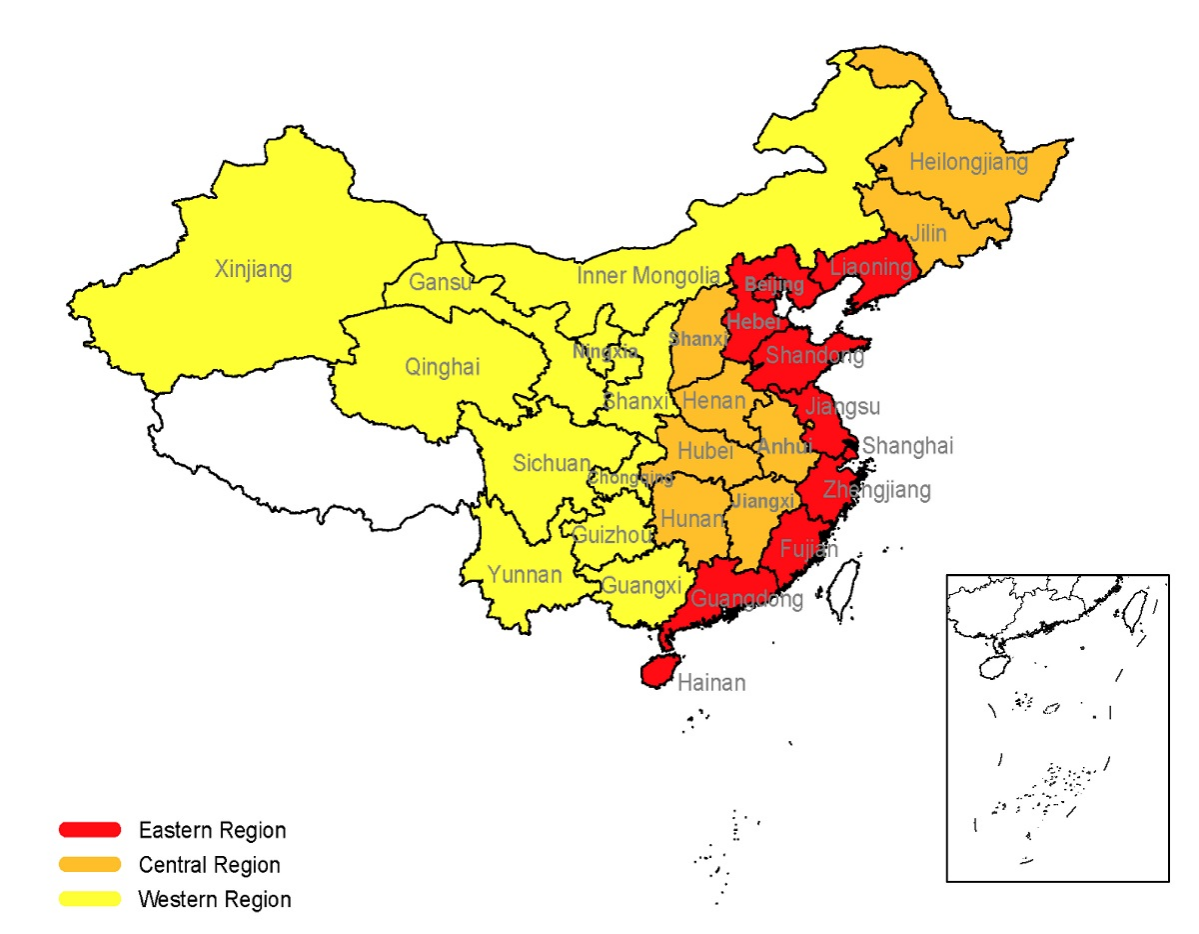
Distribution of the survey coverage areas in China.

The Chinese hospitals are divided into 3 levels: tertiary hospitals, secondary hospitals, and primary hospitals. The tertiary hospitals are the main providers of telemedicine services. Therefore, investigation of the implementation and application of telemedicine in tertiary hospitals can help in understanding the development of telemedicine in China. A web-based questionnaire survey (Multimedia Appendix 1) was conducted from August to October 2018 in Chinese tertiary hospitals that carried out telemedicine services through the Telemedicine Information Professional Committee of China (TIPC), and the survey content was with regard to telemedicine services in tertiary hospitals in 2017. TIPC is a professional organization in the telemedicine industry, gathering telemedicine practitioners and hospital administrators from different provinces in China. The survey coverage areas are shown in Figure 1.

The preliminary questionnaire was developed based on a literature review [3,14,19,25] and expert consultation combined with telemedicine practice. Ten experts with more than 5 years of working experience in telemedicine were consulted on the questionnaire design. The questionnaire was modified and optimized later according to the presurvey conducted in 18 tertiary hospitals in the Henan province. The questionnaire mainly consisted of 3 parts: telemedicine implementation, telemedicine application, and key factors related to telemedicine development. The human resource allocation, management, funding, network types, data storage, software, and hardware equipment were the main components of telemedicine implementation, and telemedicine application focused on service types, service quantity, charges, and service effects. The influencing factors in the survey mainly explored the key factors affecting the development of telemedicine. We conducted a sampling survey nationwide with the probability proportionate to size sampling technique. First, we calculated the total sample size by using the following formula.

n = Z_α/2_^2^p (1-p)/δ^2^, n_c_ = n/(1+n/N)

where Z_α/2_ is 1.96 at the significance level of α=.05, and p is assumed to be 0.5 with the principle of maximum population variance, indicating a more conservative sample size. δ is 0.1, representing that the margin of error is within 10%. N is 2060, denoting the total number of Chinese tertiary public hospitals in 2016. n_c_ is the number of the samples. According to the value above, we obtained n_c_ to be 92, and the final sample size was 103 with the waste rate being 10%. Second, on the basis of the number of tertiary hospitals in the eastern, central, and western regions of China in 2016, we distributed the sample sizes in different regions proportionally, that is, 49 hospitals in the eastern region, 27 hospitals in the central region, and 28 hospitals in the western region. At the same time, we identified the person in charge of the survey in each region and organized the questionnaire survey by using snowball sampling.

### Statistical Analysis

The total number of tertiary hospitals investigated was 185. However, the questionnaires from 24 hospitals were incomplete with high percentages of missing data, and only 161 questionnaires were valid, with an effective rate of 87.0%. The number of hospitals located in the eastern, central, and western region was 59, 54, and 48, respectively, accounting for 36.7%, 33.5%, and 29.8%, respectively, of the total number of the tertiary hospitals investigated in this study. Among the 161 hospitals, 137 hospitals provided telemedicine services and 111 hospitals obtained telemedicine services from other hospitals. The results of some items were invalid or missing. Thus, in the analysis of the corresponding content, the unqualified answers were processed as missing values. The quantitative data were described by mean values, while the qualitative data were described by count and percentages. The column chart, bar chart, pie chart, and radar chart were adopted to analyze the implementation and application of telemedicine using the Excel software (Microsoft Corp). Furthermore, the study applied the methods of chi-square test, two-sided *t* test, variance analysis, and nonparametric test to compare the development of telemedicine in different regions. Ordinal regression was adopted to model the dependence of the telemedicine service effect on other factors in the multivariate analysis by using the SPSS 23.0 software (IBM Corp), with the significance test level of *α*=.05.

## Results

### Human Resources and Funding

**Figure 2:**
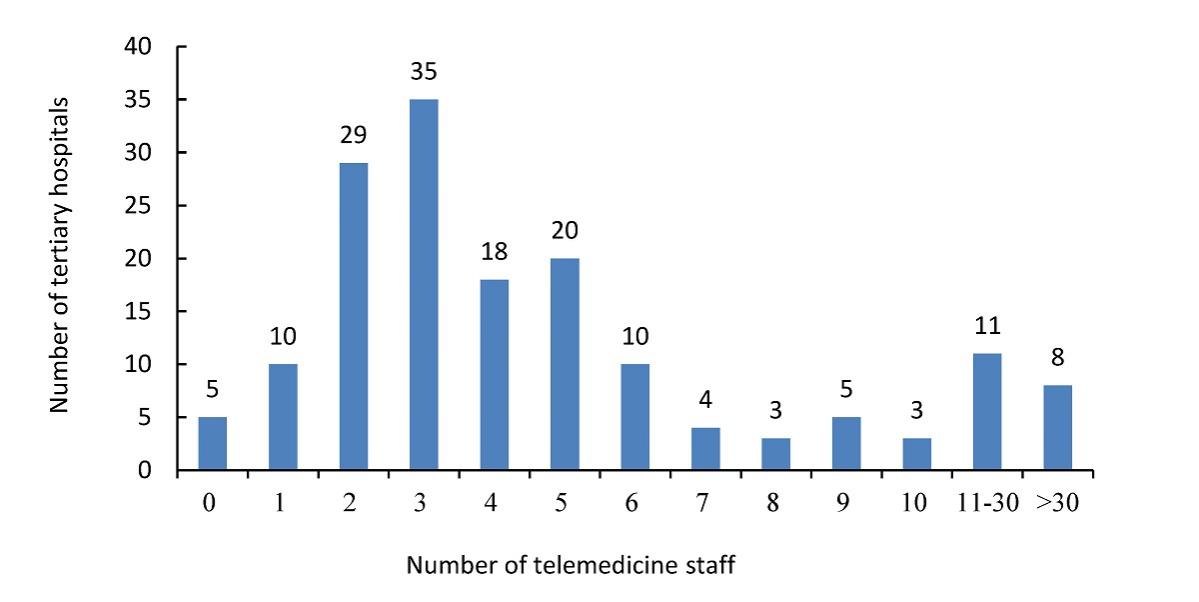
Distribution of the telemedicine staff in tertiary hospitals of China.

The number of telemedicine staff in 75.8% (122/161) of the tertiary hospitals ranged from 1 to 6 (Figure 2). Overall, the average number of telemedicine staff in each hospital was 6.8 in China, with 7.4 in the eastern region, 6.7 in the central region, and 6.2 in the western region, respectively, but there was no statistically significant difference (*P*=.83). Telemedicine staff were mainly composed of those with master’s and bachelor’s degrees. Those with a bachelor’s degree accounted for 49.58% (536/1081) of the telemedicine staff, followed by masters and junior college and below at 38.85% (420/1081) and 11.56% (125/1081), respectively. The majority of the telemedicine staff were in the fields of medicine, computer science and communication, and management (Table 1). There were statistically significant differences in the number of employees majoring in computer science and communication and medicine in different regions with *P*=.03 and *P*=.01, respectively. By contrast, no significant difference was found in the number of telemedicine staff with management majors in the different regions (*P*=.24).

**Table 1:** Educational background of the telemedicine staff in the tertiary hospitals of different regions in China (person per hospital).

Educational background	Total (n)	Eastern region (n)	Central region (n)	Western region (n)
Computer science and communication	1.8	1.4	1.6	2.5
Medicine	2.7	4.1	1.9	1.7
Management	1.3	1.3	0.9	1.6

The sources of funding for telemedicine implementation mainly include government financial support, hospital self-raising, research funding, and corporate sponsorship. According to the survey results, 83.2% (134/161) of the tertiary hospitals implemented telemedicine by self-fundraising. By comparison, only 8.1% (13/161) of the hospitals received financial support through research funding. In terms of capital investment, the proportion of tertiary hospitals with an investment of less than 500,000 RMB (approximately US $71,218) was the highest at 64.6% (104/161). Approximately 19.3% (31/161) of the hospitals invested more than 1 million RMB (approximately US $142,500) into telemedicine implementation (Table 2).

**Table 2:** Source and investment amount of telemedicine implementation in tertiary hospitals in different regions of China (N=161).

Funds			Total, N=161, n (%)	Eastern region, n=59, n (%)		Central region, n=54, n (%)		Western region, n=48, n (%)
**Sources of funds**								
	Government finance	47 (29.2)		12 (20)	18 (33)		17 (35)	
	Hospital self-raising	134 (83.2)		51 (86)	44 (82)		39 (81)	
	Research funding	13 (8.1)		7 (12)	3 (6)		3 (6)	
	Corporate sponsorship	14 (8.7)		7 (12)	5 (9)		2 (4)	
**Investment amount (RMB)^a^**								
	<100,000	59 (36.7)		21 (36)	21 (39)		17 (35)	
	100,000-500,000	45 (28.0)		17 (29)	17 (32)		11 (23)	
	500,000-1 million	26 (16.2)		10 (17)	6 (11)		10 (21)	
	1-5 million	21 (13.0)		6 (10)	7 (13)		8 (17)	
	>5 million	10 (6.2)		5 (9)	3 (6)		2 (4)	

^a^1 RMB=US $0.14.

### Management and Service Modes

As shown in Table 3, 59.1% (95/161) of the hospitals adopted a complete self-management operation pattern, while others delegated part or all of the telemedicine service to a third party. With respect to the management department, obviously, the proportions of hospitals with telemedicine administrative departments in the eastern (48/59, 81%) and western regions (34/48, 71%) were significantly higher than that in the central regions (28/54, 52%), with a significant regional difference (*P*<.001).

Telemedicine service modes include business-to-business (B2B), direct-to-consumer (DTC), and business-to-business-to-customer (B2B2C). B2B is a mode in which a medical institution provides telemedicine services to doctors in another medical institution. DTC means that medical institutions provide remote services directly to patients, while B2B2C refers to medical institutions providing telemedicine services to patients through other intermediaries such as telemedicine companies. The results suggested that 93.8% (151/161) of the tertiary hospitals carried out telemedicine services in B2B mode, which indicated the dominance of B2B mode in China. The proportion of the DTC mode (28/161, 17.4%) was closely matched to that of the B2B2C mode (32/161, 19.9%).

**Table 3:** Telemedicine management and service mode of tertiary hospitals in different regions of China (N=161).

Mode		Total, N=161, n (%)	Eastern region, n=59, n (%)	Central region, n=54, n (%)	Western region, n=48, n (%)
**Management mode**					
	Self-management mode	95 (59.1)	40 (68)	29 (54)	26 (54)
	Partial entrustment mode	51 (31.7)	16 (27)	22 (41)	13 (27)
	Complete entrustment mode	13 (8.1)	3 (5)	3 (6)	7 (15)
	Other	2 (1.2)	0 (0)	0 (0)	2 (4)
**Management department**					
	Established	110 (68.3)	48 (81)	28 (52)	34 (71)
	Being established	22 (13.7)	5 (9)	10 (19)	7 (15)
	Not established	29 (18.0)	6 (10)	16 (30)	7 (15)
**Service mode**					
	B2B^a^ mode	151 (93.8)	58 (98)	50 (93)	43 (90)
	DTC^b^ mode	28 (17.4)	13 (22)	6 (11)	9 (19)
	B2B2C^c^ mode	32 (19.9)	14 (24)	12 (22)	6 (13)

^a^B2B: business-to-business.

^b^DTC: direct-to-consumer.

^c^B2B2C: business-to-business-to-customer.

### Network Types and Security

**Figure 3:**
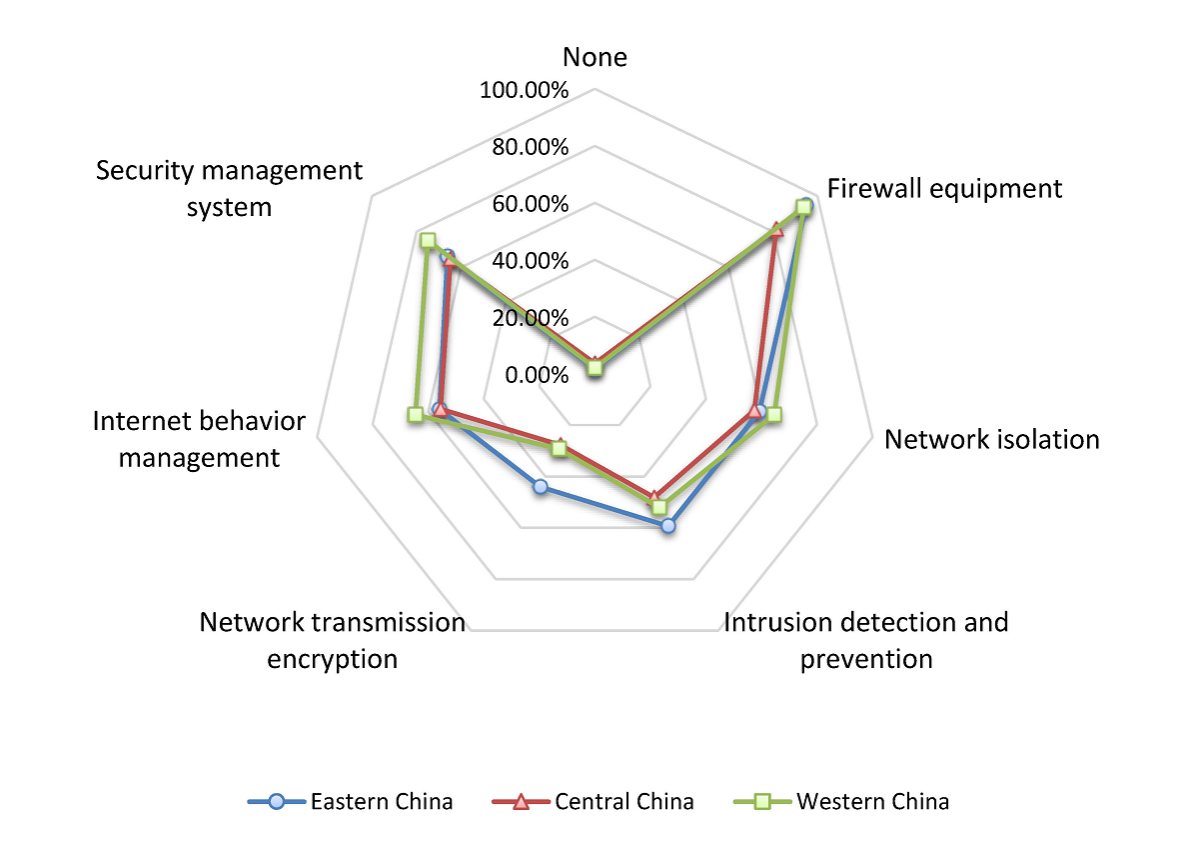
Telemedicine network security measures of tertiary hospitals in different regions of China.

Overall, the most widely used type of telemedicine network was virtual private network (VPN, a special telemedicine network constructed in China based on wired network) in 55.3% (89/161) of the hospitals, followed by internet (excluding VPN and wireless networks) in 43.5% (70/161) of the hospitals; both these networks are far ahead of 3G/4G (2/161, 1.2%) in the hospitals. In terms of regions, VPN and internet constituted the same percentage of the telemedicine networks in the central and eastern regions. Most of the hospitals (33/48, 69%) in the western region adopted VPN. With regard to the security of the telemedicine networks, overall, 97.5% (157/161) of the tertiary hospitals developed different cybersecurity measures. The proportion of the hospitals that utilized firewall equipment to ensure network security was the highest at 90.1% (145/161). The other 2 popular network security measures for telemedicine were the formulation of security management systems and the implementation of network isolation. Figure 3 illustrates the network security measures for telemedicine in each region.

### Data Storage and Security

**Figure 4:**
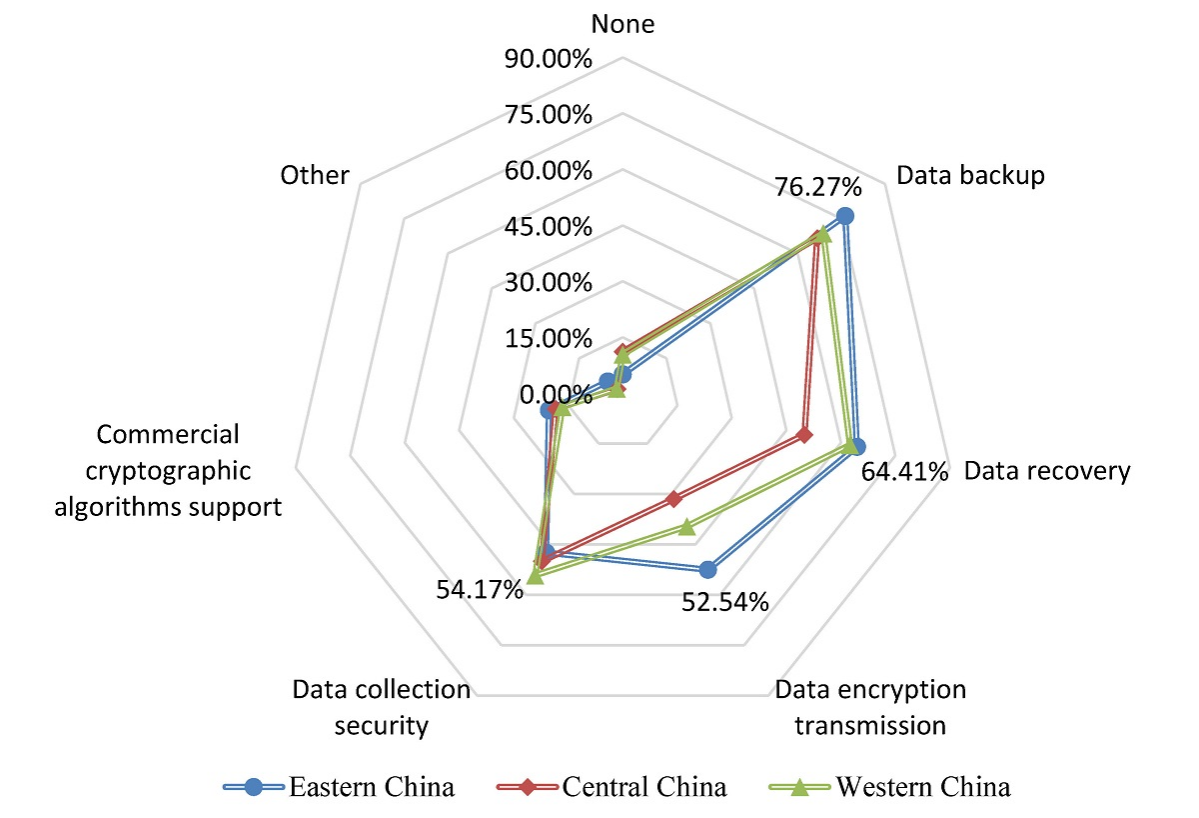
Telemedicine data security measures for tertiary hospitals in different regions of China.

Table 4 indicates how tertiary hospitals store data generated by telemedicine services. Independent storage is a relatively common method for telemedicine data storage in 36.0% (58/161) of the tertiary hospitals. For telemedicine data security measures, only a few tertiary hospitals (14/161, 8.7%) have not established security measures. Among the security measures taken by tertiary hospitals, data backup is the foremost as 70.8% (114/161) of the tertiary hospitals carried out data backup. Followed by that, 59.0% (95/161) of the hospitals implemented data recovery measures after a failure. The results in Figure 4 suggest that data backup, data recovery, and data transmission encryption are the first 3 data security measures adopted by tertiary hospitals in the eastern region. In contrast, the top 3 data security methods in the central and western hospitals are data backup, data recovery, and data collection security.

**Table 4:** Telemedicine data storage methods of tertiary hospitals in different regions of China (N=161).

Data storage methods	Total, N=161, n (%)	Eastern region, n=59, n (%)	Central region, n=54, n (%)	Western region, n=48, n (%)
Independent storage	58 (36.0)	18 (31)	23 (43)	17 (35)
Sharing with other departments	45 (28.0)	19 (32)	14 (26)	12 (25)
Sharing with other hospitals	13 (8.1)	6 (10)	4 (7)	3 (6)
No storage	31 (19.3)	10 (17)	11 (20)	10 (21)
Other	14 (8.7)	6 (10)	2 (4)	6 (13)

### Hardware and Software Equipment

**Figure 5:**
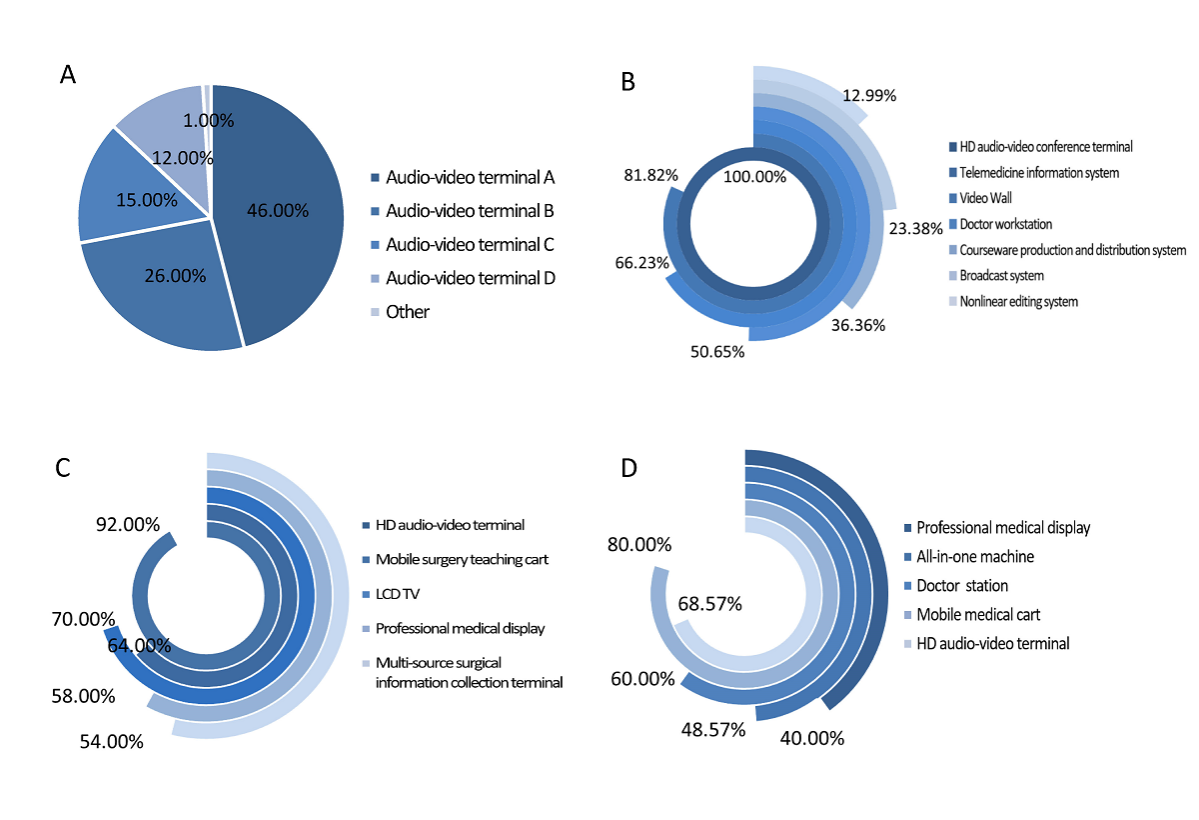
Hardware and software equipment for telemedicine services in the tertiary hospitals of China. A. Types of high-definition (HD) audio and video terminals for teleconsultation in tertiary hospitals; B. Hardware and software device configuration for remote education in tertiary hospitals; C. Hardware device configuration for remote surgery teaching in tertiary hospitals; D. Hardware device configuration for remote ward rounds in tertiary hospitals.

Of the 161 hospitals in the survey, 100 (62.1%) conducted remote videoconferencing supported by hardware for telemedicine, while other hospitals chose remote videoconferencing supported by software. The high-definition audio and video terminals were the most critical devices for teleconsultation with hardware videoconferencing, which were classified into 4 categories, that is, audio-video terminals type A, type B, type C, and type D, according to the Technical Guide for the Construction of Telemedicine Information System (2014 edition) [25]. As shown in Figure 5A, terminal type A (large multiscreens with the highest level of audio and video quality) was the mainstream, which was employed by 46.0% (46/100) of the hospitals, such as the Huawei telepresence conference system. Terminal type B (separate screen and camera) was the second most used at a usage rate of 26.0% (26/100), followed by integrated terminal type C (integrated codec and multiple audio and video interfaces) with a usage rate of 15.0% (15/100). Terminal type D (integrated camera and microphone) was used by 12.0% (12/100) of the hospitals.

Seventy-seven hospitals built remote education systems and were equipped with related hardware and software devices. The results showed that all of these hospitals were equipped with high-definition audio and videoconference terminals. Video walls and doctor workstations were also favored by many hospitals, which were present in 66% (51/77) and 51% (39/77) of the hospitals, respectively (Figure 5B). Figure 5C shows the equipment configuration for remote surgery teaching in 50 tertiary hospitals, in which 92% (46/50) of the hospitals were equipped with high-definition audio and video terminals. The usage rate of liquid-crystal display televisions (35/50, 70%) was higher than that of mobile surgery teaching carts (32/50, 64%). Only 35 tertiary hospitals developed remote ward rounds systems. The usage rate of mobile medical carts (28/35, 80%) overtook that of other devices for remote ward rounds.

### Telemedicine Services

**Figure 6:**
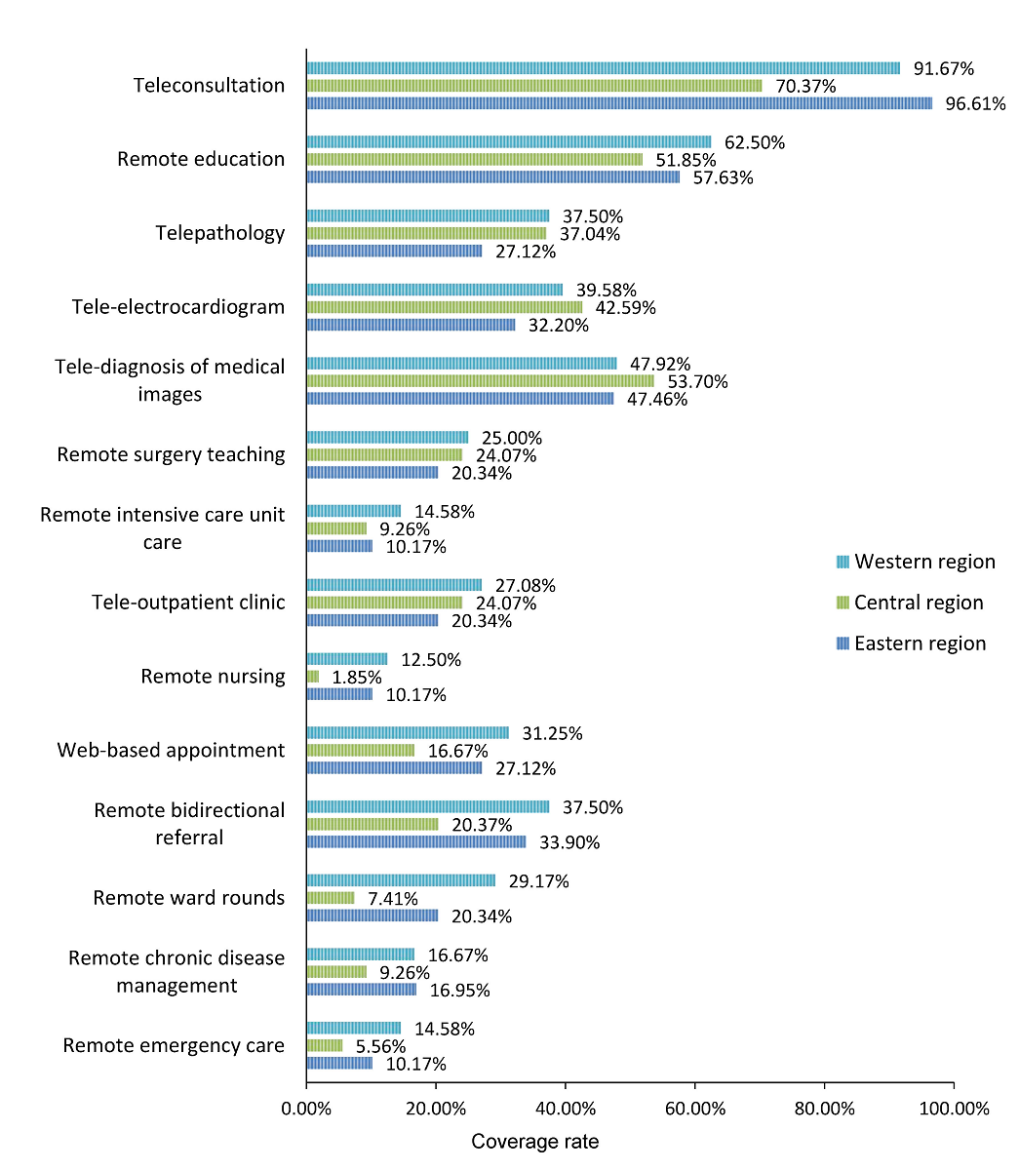
Development of various telemedicine services in the tertiary hospitals in different regions of China.

Different types of telemedicine services have been more or less implemented nationwide. The top 5 service types are teleconsultation, remote education, telediagnosis of medical images, tele-electrocardiography, and telepathology, with coverage rates of 86.3% (139/161), 57.1% (92/161), 49.7% (80/161), 37.9% (61/161), and 33.5% (54/161), respectively. Other telemedicine services such as remote intensive care unit care (18/161, 11.2%), remote nursing (13/161, 8.1%), and remote emergency care (16/161, 9.9%) were implemented relatively rarely. Figure 6 presents the types of telemedicine services in tertiary hospitals in different regions. The proportions of hospitals that conducted teleconsultations and remote ward rounds in the eastern and western regions were significantly higher than those in the central region (*P*<.001 and *P*=.02 respectively). However, there were no statistically significant differences in the proportions of the tertiary hospitals that carried out other types of telemedicine services in different regions (*P*>.05).

**Figure 7:**
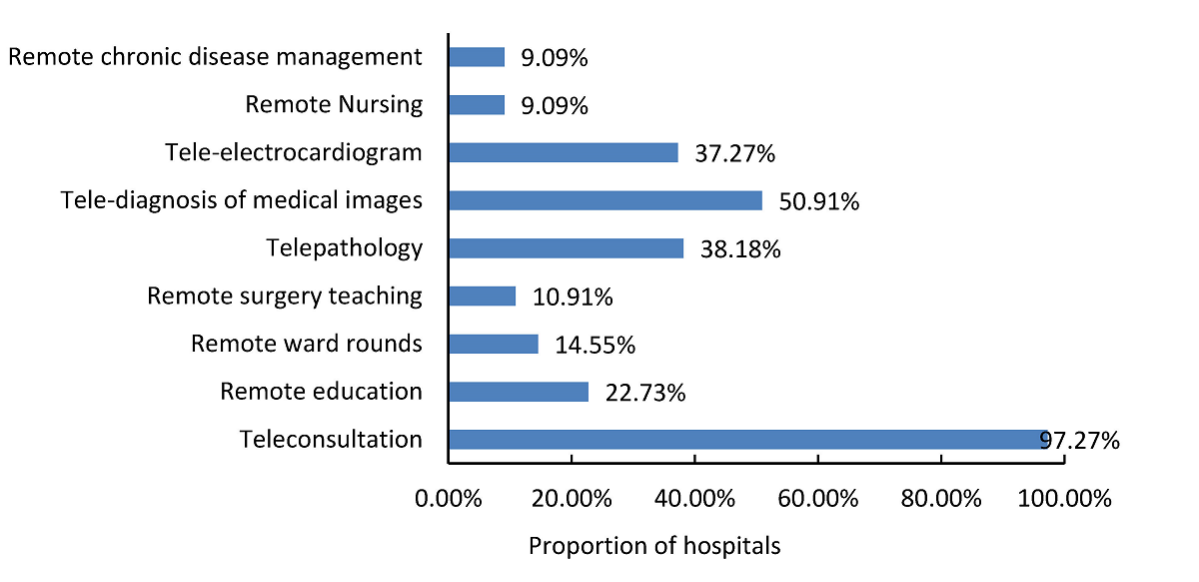
Proportion of hospitals charging for different telemedicine services.

Table 5 shows the annual service quantity of 5 major telemedicine services in tertiary hospitals, in which the annual business volume of tele-electrocardiography ranked first. The annual service volumes of teleconsultation, remote education, and telediagnosis of medical images in the western region substantially outnumbered that in eastern and central regions. As for the telemedicine charge, overall, 71.7% (114/159) of the hospitals charged for telemedicine services in the survey. However, according to the medical insurance policies in different regions, only 22.8% (26/114) of the hospitals have included telemedicine charges into medical insurance. Teleconsultation and telediagnosis (telepathology, telediagnosis of medical images, and tele-electrocardiography) were the main chargeable items for telemedicine as indicated in Figure 7.

**Table 5:** Business volume of the major telemedicine services in tertiary hospitals (cases per hospital) in different regions of China.

Telemedicine service	Total (n)	Eastern region (n)	Central region (n)	Western region (n)
Teleconsultation	714	604	532	1045
Remote education	44	27	35	76
Telepathology	139	48	266	107
Tele-electrocardiography	3342	2645	4123	3292
Telediagnosis of medical images	1107	593	405	2443

**Figure 8:**
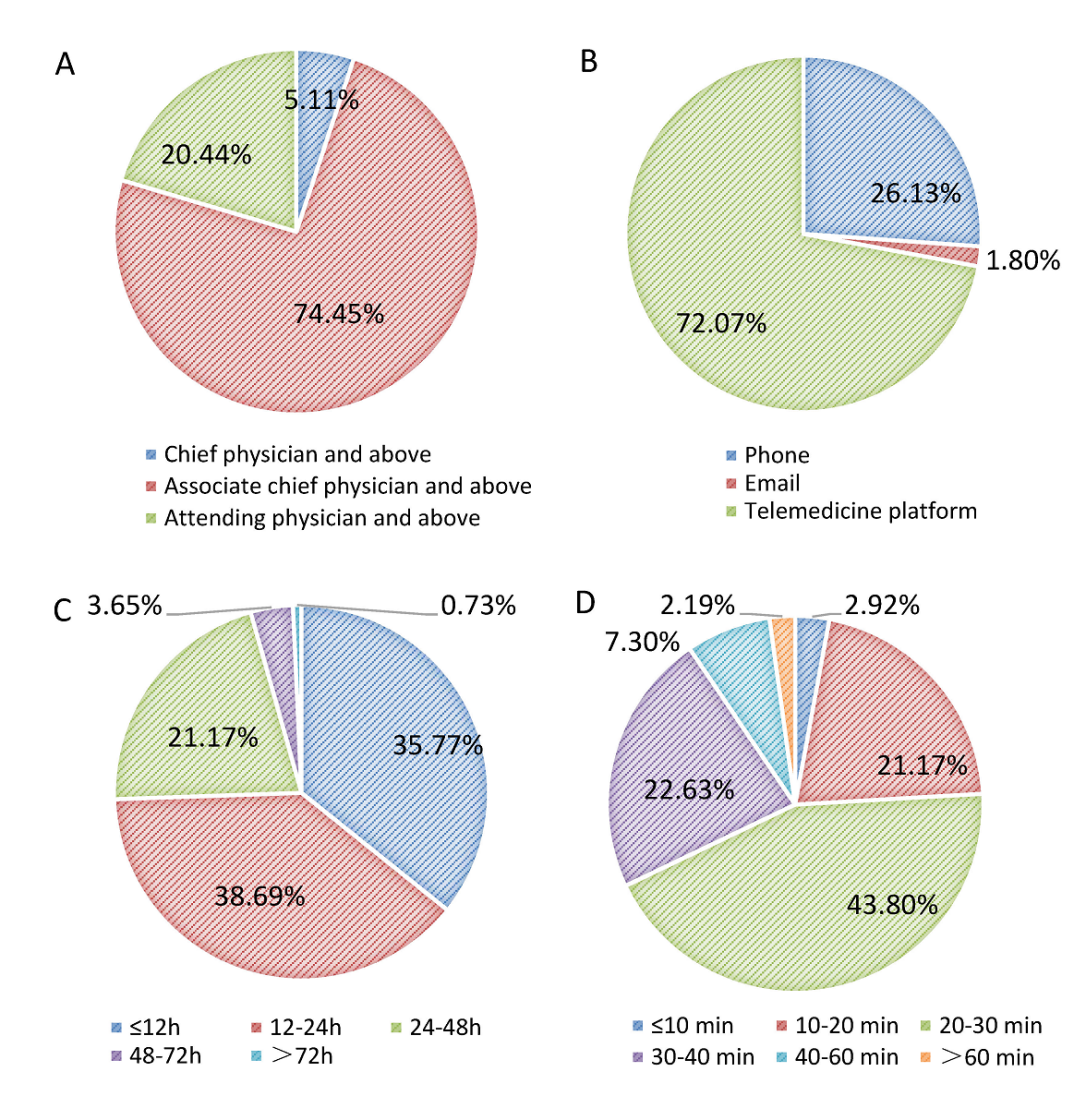
Teleconsultation status in tertiary hospitals: A. Levels of teleconsultation specialists; B. Ways to apply for teleconsultation; C. Average waiting time for teleconsultation; D. Average duration of teleconsultation cases.

Teleconsultation, as the core service of telemedicine, needs to be explored in depth. Figure 8A depicts that the level of medical experts providing teleconsultation services was deputy chief physician and above in most tertiary hospitals (109/137, 79.6%). Figure 8B illustrates that 72.1% (80/111) of the tertiary hospitals applied for teleconsultation through the telemedicine platform, which was the most important way. In terms of the response duration of teleconsultation, that is, the time interval from the consultation application to the start of the consultation, 74.5% (92/137) of the hospitals had a waiting time within 24 hours. Figure 8D shows that the average duration of each teleconsultation in most hospitals ranged from 10 to 40 minutes. Among them, the percentage of teleconsultations lasting 20-30 minutes was the highest in 43.8% (60/137) of the hospitals. As far as the effect of teleconsultation was concerned, the proportion of hospitals that considered the effect as good and excellent was 51.4% (57/111) and 31.5% (35/111), respectively, whereas 17.1% (19/111) of the hospitals believed the consultation effect to be fair or poor. Remote education is another core business of telemedicine. The overall participation frequency of remote education in tertiary hospitals was relatively low; 83.8% (93/111) of the hospitals performed 0-6 instances of remote education every month. The proportion of hospitals participating in remote education with a frequency of 7-10 cases, 11-14 cases, and 15 cases and above every month was 6.3% (7/111), 1.8% (2/111), and 8.1% (9/111), respectively. As for the effect of remote education, 73.0% (81/111) of the tertiary hospitals believed that it had a certain effect on improving the medical service level, and 26.1% (29/111) of the hospitals thought it had a great effect. However, 0.9% (1/111) of the hospitals considered that it had no effect.

### Key Factors Influencing Telemedicine Development

**Figure 9:**
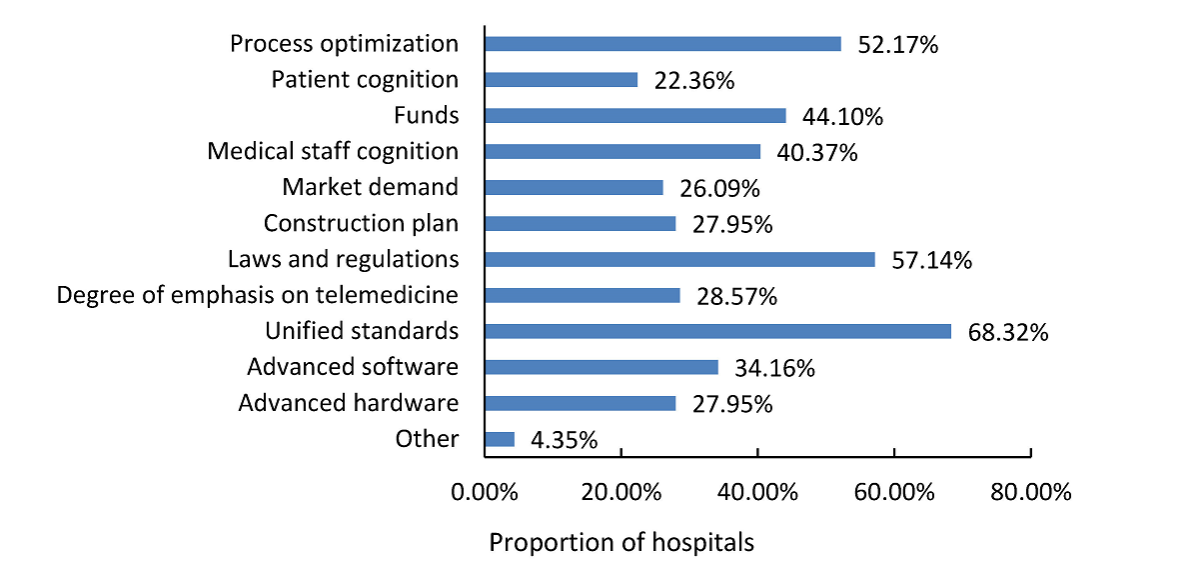
Key factors affecting the development of telemedicine in tertiary hospitals of China.

Standard formulation was the most crucial factor, as 68.3% (110/161) of the tertiary hospitals believed that the lack of uniform standards hindered the promotion of telemedicine in hospitals. The other influencing factors of the development of telemedicine in tertiary hospitals are listed in Figure 9. In order to explore the key factors influencing the effects of telemedicine further, we considered the core telemedicine services (ie, teleconsultation and remote education) as examples and applied ordinal logistic regression to analyze the relationship between the effect of telemedicine and the aforementioned factors such as human resources, funding, region, management, and service modes. The effect of teleconsultation refers to how useful it is for the treatment and health of the patient. Thus, the teleconsultation effect was divided into 3 categories (1=poor or fair, 2=good, and 3=excellent). The effect of remote education was expressed in terms of improvement in the medical service level (1=no improvement, 2=certain improvement, 3=great improvement), in which “certain improvement” means that remote education helps to improve the medical service level of the hospital, but the improvement is not significant. Taking the high-level effect as a reference, as many factors as possible were included in the models. Some results are presented in Table 6 and Table 7. The complete parameter estimation results are shown in the Multimedia Appendix 2 and Multimedia Appendix 3.

As shown in Table 6, an increase in the number of medical professionals may have a positive impact on the teleconsultation effect (*P*=.04). The adoption of the DTC mode, the support of scientific research funds, and the charging for consultation services could improve the teleconsultation effect, with *P*=.003, *P*=.01, and *P*<.001, respectively. Compared with 3G/4G, VPN and internet could significantly improve the consultation effect (*P*=.02 and *P*=.02, respectively). No data storage would reduce the consultation effect compared with other data storage methods, as no statistical significance was found (*P*=.07). Sharing data with other hospitals could significantly improve the consultation effect (*P*=.04). As the duration of the consultation increased, the consultation effect decreased to varying degrees. High-level experts were helpful in improving the consultation effect (*P*=.03).

Similarly, the adoption of the DTC mode, the provision of research funding, and the charging for service had a positive impact on remote education by improving the medical service level. However, regional differences had no impact on the effect of remote education. Compared with hospitals that did not establish professional management departments, the remote education effects of hospitals that were establishing departments or with already established departments were better, with *P=*.04 and *P*=.25, respectively. Compared with the 0-3 instances of remote education every month, the frequency of 15 instances and above every month could significantly improve the effect of remote education (*P*=.01).

For this study, the Checklist for Reporting Results of Internet e-Surveys has been uploaded in Multimedia Appendix 4.

**Table 6:** Ordinal logistic regression results of the influencing factors of the consultation effect.

Variable			Regression coefficient	Standard error	Wald statistic	*P* value
**Educational background of the professionals**						
	Computer science and communication professionals		–0.31	0.19	2.63	.11
	Medical professionals		0.09	0.04	4.09	.04
	Management professionals		–0.27	0.14	3.87	.049
**Service mode**						
	**B2B^a^mode**					
		Yes	1.25	2.45	0.26	.61
		No^b^	N/A^c^	N/A	N/A	N/A
	**DTC^d^mode**					
		Yes	3.54	1.20	8.68	.003
		No	N/A	N/A	N/A	N/A
	**B2B2C^e^mode**					
		Yes	–0.57	1.15	0.25	.62
		No	N/A	N/A	N/A	N/A
**Investment amount (RMB)^f^ (reference=less than 100,000 RMB)**						
	>5 million		–1.26	1.57	0.65	.42
	1-5 million		0.01	1.04	0.00	.99
	500,000-1 million		–3.26	1.25	6.79	.01
	100,000-500,000		–0.61	0.85	0.52	.47
**Funds**						
	**Government financial support**					
		Yes	–0.70	0.92	0.58	.45
		No	N/A	N/A	N/A	N/A
	**Hospital self-raising**					
		Yes	–1.47	1.17	1.57	.21
		No	N/A	N/A	N/A	N/A
	**Research funding**					
		Yes	3.72	1.51	6.09	.01
		No	N/A	N/A	N/A	N/A
	**Corporate sponsorship**					
		Yes	–0.18	1.34	0.02	.89
		No	N/A	N/A	N/A	N/A
**Network types (reference=3G/4G)**						
	Virtual private network		4.55	2.02	5.08	.02
	Internet		4.75	2.00	5.67	.02
**Data storage (reference=other)**						
	Independent storage		1.41	1.22	1.34	.25
	Sharing with other departments		–0.16	1.29	0.02	.90
	Sharing with other hospitals		3.66	1.79	4.19	.04
	No storage		–2.43	1.32	3.40	.07
**Expertise level (reference=attending physician and above)**						
	Chief physician and above		5.29	2.37	4.99	.03
	Associate chief physician and above		1.01	0.82	1.51	.22
**Duration of consultation per case (reference≤10 min)**						
	60 min		–11.10	3.50	10.06	.002
	40-60 min		–5.50	2.96	3.45	.06
	30-40 min		–7.83	3.05	6.61	.01
	20-30 min		–6.08	2.89	4.42	.04
	10-20 min		–3.37	2.66	1.61	.21
**Charge**						
	Yes		4.26	1.02	17.30	<.001
	No		N/A	N/A	N/A	N/A

^a^B2B: business-to-business.

^b^Reference.

^c^N/A: not applicable.

^d^DTC: direct-to-consumer.

^e^B2B2C: business-to-business-to-customer.

^f^1 RMB=US $0.14.

**Table 7:** Ordinal logistic regression results of the influencing factors of the remote education effect.

Variable				Regression coefficient		Standard error		Wald statistic		*P* value
**Region (reference=center)**										
	East		–1.04		1.10		0.90		.34	
	West		–1.66		1.16		2.03		.15	
**Service mode**										
	**B2B^a^mode**									
		Yes	5.70		4.68		1.48		.22	
		No^b^	N/A^c^		N/A		N/A		N/A	
	**DTC^d^mode**									
		Yes	4.01		1.59		6.39		.01	
		No	N/A		N/A		N/A		N/A	
	**B2B2C^e^mode**									
		Yes	–2.57		1.47		3.05		.08	
		No	N/A		N/A		N/A		N/A	
**Professional management department (reference=not established)**										
	Established		1.45		1.27		1.31		.25	
	Being established		3.67		1.78		4.28		.04	
**Investment amount (RMB)^f^ (reference=less than 100,000 RMB)**										
	>5 million		0.60		2.26		0.07		.79	
	1-5 million		0.19		1.48		0.02		.90	
	500,000-1 million		–2.22		1.82		1.48		.22	
	100,000-500,000		0.39		1.12		0.12		.73	
**Funds**										
	**Government financial support**									
		Yes	–2.22		1.22		3.31		.07	
		No	N/A		N/A		N/A		N/A	
	**Hospital self-raising**									
		Yes	–2.18		1.53		2.03		.15	
		No	N/A		N/A		N/A		N/A	
	**Research funding**									
		Yes	4.69		1.83		6.61		.01	
		No	N/A		N/A		N/A		N/A	
	**Corporate sponsorship**									
		Yes	–1.77		1.65		1.15		.28	
		No	N/A		N/A		N/A		N/A	
**Frequency of remote education (reference=0-3 times a month)**										
	15 instances and above per month		5.95		2.11		7.94		.01	
	11-14 instances per month		–3.64		6.51		0.31		.58	
	7-10 instances per month		2.99		1.69		3.13		.08	
	4-6 instances per month		0.14		1.11		0.02		.90	
**Charge**										
	Yes		4.30		1.53		7.97		.01	
	No		N/A		N/A		N/A		N/A	

^a^B2B: business-to-business.

^b^Reference.

^c^N/A: not applicable.

^d^DTC: direct-to-consumer.

^e^B2B2C: business-to-business-to-customer.

^f^1 RMB=US $0.14.

## Discussion

### Principal Findings

Based on a national survey, this study analyzed the development of telemedicine in Chinese hospitals from multiple aspects, including implementation, application, and the key factors influencing telemedicine service effects. We found that telemedicine services were mainly carried out in the form of hardware videoconferences in B2B mode through VPN in Chinese tertiary hospitals, with various service types and a large service quantity, which were considered to have positive effects on the improvement of medical treatment in primary hospitals. Despite the rapid development of telemedicine in China, there are still problems such as the lack of uniform standards and laws, which reminds us that a lot of work is needed to improve the standardization of telemedicine services and establish legal protection for telemedicine services.

In terms of human resource and management, 31.7% (51/161) of the tertiary hospitals had no specific management departments, and some tertiary hospitals did not even have full-time staff. Further, the number of telemedicine staff with majors in computer science and communication and management was relatively small. As a burgeoning, cutting-edge, and multidisciplinary technology, telemedicine is in high need of a compound talent team that integrates the knowledge structure of science, engineering, and medicine. According to the technical guide of telemedicine in China, a tertiary hospital should set up an independent telemedicine department with medical, information technology, and management professionals and technical personnel [25]. However, many hospitals do not meet the requirements of the technical guide; therefore, more work is still needed to improve the organization of a telemedicine management department. Previous studies have suggested that sufficient capital investment and leadership attention are the primary factors affecting the development of remote education and remote pathology diagnosis [26]. In China, 35.4% (57/161) of the hospitals invest more than 500,000 RMB (approximately US $71,218) per year, and 28.0% (45/161) of the hospitals invest 100,000-500,000 RMB (approximately US $14,244-$71,218) per year, thereby providing financial support for telemedicine development. Government investments and hospital self-raising were the main sources of funds, which were consistent with the results of another telemedicine survey [27], thereby revealing the national attention on telemedicine.

Through the investigation, we found that B2B was the main telemedicine service mode in China. Historically, as the original intention of telemedicine is that experts in tertiary hospitals assist doctors in secondary or primary hospitals at different locations to provide solutions to the management of complicated diseases by videoconferencing, telemedicine services are still mainly limited to the B2B mode, and the DTC mode remains to be improved [28,29]. However, with the development of medical information, the DTC mode is considered to be a further developmental tendency for telemedicine [30,31], which will enable the patients to apply for telemedicine services with greater accessibility and convenience, such as choosing the doctors they prefer [32]. In 2018, a policy was issued in China to allow medical institutions to set up internet hospitals and to directly carry out telemedicine services for revisiting patients with common and chronic diseases. To date, about 158 internet hospitals in China have been built to provide web-based telemedicine consultations directly to the patients [33]. We believe that the telemedicine service mode will change from B2B to DTC in the near future.

According to the results of the network construction, VPN was the main type of network used for telemedicine. With the advantages of high security, flexible access, and low latency [34], VPN is the preferred network for telemedicine in the technical guide [25]. However, 44.7% (72/161) of hospitals still do not have access to VPN. A policy was introduced in China in 2018 to encourage telecom enterprises to provide high-quality dedicated internet access and VPN for medical institutions and to promote the construction of dedicated networks for telemedicine and guarantee the quality of medical-related data transmission services. It can be predicted that private networks will be the predominant type of network used for telemedicine services in the future. Telemedicine provides more chances for accessing patients’ personal information. However, this is accompanied by a threat to the security of the medical data [35]. Therefore, security measures should be formulated in the implementation of telemedicine. In China, 91.3% (147/161) and 97.5% (157/161) of the tertiary hospitals have established data security measures and network security measures, respectively, such as firewall, network isolation, and internet behavior supervision, thereby reflecting their emphasis on information security.

The survey results showed that 62.1% (100/161) of the hospitals conducted hardware videoconferences instead of software videoconferences for teleconsultation. Despite the availability of low-cost videoconferencing software, due to the high requirements for information transmission and video quality, many hospitals have adopted high-performance hardware videoconferencing owing to the advantages of high definition, security, stability, and interoperability [36]. In 2017, an average of 714 remote consultations were conducted in each tertiary hospital, indicating a huge increase compared to the number of teleconsultations in 2014 (no more than 7 times per quarter) [27]. For different types of remote diagnosis carried out in tertiary hospitals, the service quantity of tele-electrocardiography diagnosis ranked first, with an average number of 3342 cases in each hospital, which was related to the relatively low requirements on equipment and network speed [37]. Unlike the remote consultation and diagnosis, the application of telemedicine is relatively limited in intensive care and nursing and emergency care because of the immature technology, lack of legislation on charging standards and responsibility distinction, and the low acceptance by patients and medical staff [27,38,39].

Studies have revealed that the lack of uniform standards and laws was an essential factor hindering the development of telemedicine, which might lead to repeated and chaotic implementation of telemedicine [40]. Some scholars believe that since physical examination cannot be performed in telemedicine, medical safety cannot be guaranteed, and the responsibilities for the privacy and safety of patients are not clearly assigned to the doctors participating in telemedicine services [41-43]. Therefore, new legislation on telemedicine should be introduced to guide the implementation of telemedicine and assign the medical responsibilities clearly. Another factor hindering the development of telemedicine is the imperfect medical insurance system. In the United States of America, the proportion of medical insurance payment for telemedicine is 0%-67% [44], whereas in China, only some provinces such as Guizhou and Sichuan realize the medical insurance reimbursement. The government should formulate a medical insurance policy as well as develop a reasonable benefit distribution mechanism to promote the sustainable implementation of telemedicine in China.

This study shows that most hospitals believe that telemedicine is effective and could improve the medical service level of the hospitals, which was consistent with the results of many studies that positively evaluated telemedicine [45,46]. A study showed that as the frequency of consultation increased, primary care providers could significantly improve knowledge acquisition [47]. The DTC mode, support of scientific research funds, and service charges had a significant impact on the effects of both teleconsultation and remote education. Compared to the mainstream B2B mode, the obvious feature of the DTC mode is the participation of patients, which may help to obtain more accurate patient information during the consultation process and allow patients to interact with doctors. The supports for research funding and service charges can significantly increase the enthusiasm of doctors. With the encouragement of the government, many provinces in China have issued pricing and payment policies for telemedicine services. As a result, the charges for telemedicine services will be rule-based, more standardized, and reasonable in the future.

### Comparison With Prior Work

Although previous studies have analyzed the utilization of telemedicine in a certain region, the overall development of telemedicine in China has not yet been studied, especially in terms of network construction, security measures, and hardware and software facilities. This study investigated the implementation and application of telemedicine from a national perspective, which will provide people with a comprehensive, multilevel, and multifaceted understanding of telemedicine development in China. Moreover, to our knowledge, this is the first study to use ordinal regression models to deeply analyze the factors influencing the effectiveness of telemedicine in multiple dimensions, including human resources, funding, management and service modes, networks, and charging, which will supply a reference for telemedicine planning.

### Limitations

Although our findings provide a deep insight into the development of telemedicine in China, this study has several limitations. As the first nationwide survey on telemedicine in China, our sample is nationally representative and covers most areas of China. However, the sample size is still insufficient, as some areas with few tertiary hospitals were not included, such as Tibet; therefore, the scope of the research needs to be further expanded. Besides, we have only considered tertiary hospitals as the survey object; however, patients’ attitude toward telemedicine is also important for the development of telemedicine, which will be the focus of the next study.

### Conclusions

We conducted a quantitative analysis of the overall implementation and application of telemedicine in China with the data from 161 tertiary hospitals in 29 provinces, autonomous regions, and municipalities. Our findings revealed that telemedicine services were carried out in most parts of China, and most tertiary hospitals provided telemedicine services in B2B mode through the hardware videoconferencing. VPN was the most widely used type of telemedicine network, and audio-video terminals with large screens was the mainstream hardware. Teleconsultation, remote education, and telediagnosis were the main types of telemedicine service. Service modes, financial sources, network types, service charges, and medical experts are the main factors influencing the effect of teleconsultation and remote education. The management, uniform standards, and legislation still need to be improved for the sustainability of telemedicine in China. To plan the development of telemedicine further, our research provides a reference for policymakers to promote the implementation of DTC mode of telemedicine, expand the coverage of VPN, develop innovative service patterns such as remote nursing and remote care in intensive care units, and formulate complete telemedicine laws and regulations.

## Appendix

Multimedia Appendix 1 Web-based questionnaire survey (translated version).

Multimedia Appendix 2 Estimation results of ordinal regression analyzing the influencing factors of teleconsultation.

Multimedia Appendix 3 Estimation results of ordinal regression analyzing the influencing factors of remote education.

Multimedia Appendix 4 Data reporting guidelines and checklist for reporting results of internet e-surveys (CHERRIES).

## Abbreviations

B2B: business-to-business
B2B2C: business-to-business-to-customer
DTC: direct-to-consumer
TIPC: Telemedicine Information Professional Committee of China
VPN: virtual private network
