# Supplementary material for: Implementation and Application of Telemedicine in China: Cross-Sectional Study
Source: JMIR Mhealth Uhealth. 2020 Oct 23;8(10):e18426. doi: 10.2196/18426 (PMC7647817; doi:10.2196/18426)
Supplement: Multimedia Appendix 1 [file mhealth_v8i10e18426_app1.pdf]

Survey on the Development of Telemedicine in Chinese Tertiary Hospitals

(part of translation version)

Introduction

The survey was organized by the Telemedicine Informationization Professional Committee of China (TICP), with the aims to fully understand the development of telemedicine in China, and provide a scientific basis for the further development of telemedicine. We sincerely hope all participating hospitals provide accurate information. We will take any valuable information provided seriously and pay attention to protecting the hospital information, ensuring that the information will not be leaked.

Notes on filling the questionnaire

1.The person filling in the questionnaire should be an employee responsible for telemedicine work.
2. The content of the questionnaire survey occurred from January 1, 2017 to December 31, 2017.

Hospital name： ______________________________

Basic information

A1.What is the management mode of telemedicine service in the hospital?
1. Self-management mode
2. Partial entrustment mode
3. Completely entrust mode
4. Other

A2. What is the service mode of telemedicine in the hospital?
1. B2B mode
2. DTC mode
3. B2B2C mode
4. Other

A3.How much money was invested in the construction of telemedicine in the hospital in 2017?
1. <100,000 RMB

2. 100,000~500,000 RMB
3. 500,000~1 million RMB
4. 1~5 million RMB
5. >5 million RMB

A4. What is the sources of funds used in the construction of telemedicine in the hospital? (multiple choices)

1. Government finance
2. Hospital self-raising
3. Research funding
4. Corporate sponsorship
5. Other

A5. Has the hospital set up a special operation and management department for telemedicine business?
1. Having already set up a management department
2. Preparing to set up a management department
3. No plans to set up a management department

A6. How many staff are engaged in telemedicine in the hospital?
Please fill in the number of staff_______

A7.What are the specialties of the staff engaged in telemedicine in the hospital?
1. Computer science and communications, please fill in the number of staff_______
2. Medicine, please fill in the number of staff_______
3. Management, please fill in the number of staff_______
4. Other specialty, please fill in the number of staff_______

A8. What are the qualifications of the staff engaged in telemedicine in the hospital?
1. Master degree or above, please fill in the number of staff_______
2. Bachelor degree, please fill in the number of staff_______
3. College degree or below, please fill in the number of staff_______

Implementation of telemedicine

B1. What kind of network does your hospital use for telemedicine?
1. Virtual private network (VPN, constructed based on wired network), with a bandwidth of ____________
2. Public Internet(excluding VPN and wireless networks), with a bandwidth of _____
3. 3G/4G, with a bandwidth of ____________

B2. What methods does your hospital adopt to ensure network security (multiple

choices)
1. None
2. Firewall equipment
3. Network isolation
4. Intrusion detection and prevention
5. Network transmission encryption processing
6. Internet behavior management
7. Security management system
8. Other

B3. Which kind of data storage method for telemedicine is adopted by your hospital?
1. Independent storage (independent telemedicine data center or computer room)
2. Sharing with other departments of the hospital
3. Sharing with other hospitals
4. Not storage
5. Others (please specify) _______________

B4. What data security measures have been taken by your hospital (multiple choices）

1. None
2. Safe and complete data backup
3. Data recovery
4. Data encryption transmission
5. Data collection a security
6. Support the commercial cryptographic algorithm
7. Other

B5. Which of the following methods is used for teleconsultation in your hospital?

1. Software video conference（using computer and video conference software）, the software used is ___________________

2. Hardware video conference（video communication method based on embedded architecture）

3. Email
4. Telephone
5. Others (please specify) _________________

B6. If your hospital uses hardware video conferencing, what is the highest-level audio-visual terminal used? And the audio-visual terminal manufacturer is________________.

1. Audio-video terminal A 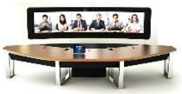

2. Audio-video terminal B 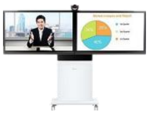

3. Audio-video terminal C 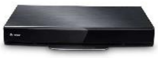

4. Audio-video terminal D 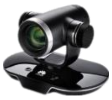

5. Other

B7. What telemedicine systems have been established in your hospital currently (multiple choices)?
1. Teleconsultation system, manufacturer is ___________ (If you choose 1, please answer B8)
2. Remote education system (if you choose 2, please answer B9)
3. Remote ward rounds system (if you choose 3, please answer B10)
4. Remote surgery teaching system (if you choose 4, please answer B11)
5. Remote pathology system
6. Remote image diagnosis system
7. Remote ECG diagnosis system
8. Tele-outpatient clinic system
9. Remote emergency care system
10. Remote Chinese medicine system
11. Other

B8. Is your hospital's LIS, PACS and other information systems connected to the teleconsultation system through interface modification?(to realize mutual inquiry, browse, record and use patient medical records, teleconsultation results, referral appointments, image ECG data, etc.)
1. Yes
2. No

B9. What software and hardware equipment does your hospital have for distance education (multiple choices)?
1. High-definition video and audio conference terminal
2. Video wall
3. Telemedicine information system
4. Doctor workstation (providing doctors with information viewing and browsing services, etc.)
5. Non-linear editing system

6. Broadcast system
7. Courseware production and release system
8. Others (please specify) ___________________

B10. What equipment does your hospital have for remote ward rounds (multiple choices)?
1. High-definition video and audio terminal
2. Mobile cart
3. Doctor workstation
4. All-in-one machine
5. Professional medical display
6. Others (please specify) ________________

B11. What equipment does your hospital have for remote surgical teaching?(multiple choices）?

1. High-definition video and audio terminal
2. Mobile surgical teaching cart
3. LCD TV
4. Professional medical display
5. Multi-source operation information collection terminal
6. Others (please specify) __________________

Telemedicine service applications

C1. What types of telemedicine applications have been developed in your hospital (multiple choices)?
1. Teleconsultation, the annual business volume: ________________cases per year
2. Remote education, the annual business volume: ____________cases per year
3. Telepathology, the annual business volume:_______________cases per year
4. Tele-electrocardiogram, the annual business volume:___________cases per year
5. Tele-diagnosis of medical images, the annual business volume:____________cases per year
6. Remote surgery teaching
7. Remote intensive care
8. Tele-outpatient clinic
9. Remote nursing
10. Online appointment
11.Remote bidirectional referral
12. Remote ward rounds
13. Remote chronic disease management
14. Remote emergency care
15.Other, ________

C2. What is the role of your hospital in the implementation of telemedicine?
1. Telemedicine service provider (if you choose 1, please answer Question C3-C4 and skip to Question C18 to continue)
2. Telemedicine applicants who apply for service and receive telemedicine service (if you choose 2, please skip to Question C5)
3. Both (if you choose 3, answer all the following questions)

C3. What is the average duration of teleconsultation in your hospital?
1. ≤10 minutes/case
2. 10-20 minutes/case
3. 20-30 minutes/case
4. 30-40 minutes/case
5. 40-60 minutes/case
6. >60 minutes/case

C4. What is the average waiting time for consultations in your hospital (from receipt of consultation application to the start of consultation)?
1. ≤12 hours
2. 12-24 hours
3. 24-48 hours
4. 48-72 hours
5. >72 hours

C5. How does your hospital apply for teleconsultation to the superior hospital?
1. Telephone appointment
2. Email appointment
3. SMS appointment
4. Telemedicine platform appointment
5. Others (please specify)_________

C6. What does your hospital think of the effect of teleconsultation?
1. Excellent
2. Good
3. Fair
4. Poor

C7. What is your hospital's willingness to participate in distance education?
1. Strong willingness to participate
2. Willing to participate
3. No interest

C8. How often does your hospital participate in distance education every month?
1. 0~3 times

2. 4~6 times
3. 7~10 times
4. 11~14 times or more
5. 15 times or more

C9. What does your hospital think of the effect of telemedicine education in promoting the improvement of hospital medical level?
1. No improvement
2. Certain improvement
3. Great improvement

C10.What are the charge items included in the telemedicine business currently carried out by your hospital (multiple choices)?
1. Teleconsultation
2. Remote education
3. Remote ward rounds
4. Remote surgery teaching
5. Telepathology
6. Tele-diagnosis of medical images
7. Tele-electrocardiogram
8. Remote nursing
9. Remote chronic disease management
10. Other

Future development of telemedicine

D1. What do you think are the key factors that affect the improvement of the hospital's telemedicine level (multiple choices) ?
Please sort (in descending order of importance) ______________________________
1. Advanced software
2. Advanced hardware
3. The process optimization of telemedicine services
4. unified standards and regulations
5. Enough attention from the hospital’s leaders
6. The establishment of telemedicine laws and regulations
7. Construction plan
8. Market demand
9. Cognition of medical staff
10. Sufficient funds
11. Patient cognition
12. Other

D2. What new telemedicine technology practices are being carried out in your hospital (multiple choices)?
1. Using wearable devices for remote monitoring and health management
2. Remote intelligent rounds
3. Telemedicine service based on 5G technology
4. Remote ultrasound examination
5. Combined Telemedicine with artificial intelligence diagnosis
6. Remote surgical guidance based on VR (virtual reality) technology
7. Telemedicine robot
8. Virtual doctor-patient interaction
9. Other

D3. What problems did your hospital encounter during the implementation of telemedicine? How was it resolved? Please talk about the relevant experience of your hospital.

D4. What are your views or suggestions on telemedicine?

Thank you for your cooperation!
