# Supplementary material for: Implementation and Application of Telemedicine in China: Cross-Sectional Study
Source: JMIR Mhealth Uhealth. 2020 Oct 23;8(10):e18426. doi: 10.2196/18426 (PMC7647817; doi:10.2196/18426)
Supplement: Multimedia Appendix 2 [file mhealth_v8i10e18426_app2.pdf]

Multimedia Appendix 1. Estimation results of ordinal regression analyzing the influencing factors of teleconsultation effect

		Coefficients	S.E.	Wald	df	P	95% Confidence Interval	
							Lower	Upper
Threshold	Fair or Poor	4.806	6.106	.619	1	.431	-7.162	16.774
	Good	9.885	6.256	2.497	1	.114	-2.376	22.145
location	Computer science and Communication Professionals(number)	-.309	.191	2.628	1	.105	-.683	.065
	Medical professionals(number)	.086	.043	4.090	1	.043	.003	.170
	Management professionals (number)	-.270	.137	3.866	1	.049	-.539	-.001
	**Region**							
	East	-.073	1.015	.005	1	.943	-2.063	1.917
	West	-.617	.906	.465	1	.496	-2.393	1.158
	Center	0[a]	.	.	0	.	.	.
	**B2B mode**							
	Yes	1.248	2.453	.259	1	.611	-3.560	6.056
	No	0[a]	.	.	0	.	.	.
	**DTC mode**							
	Yes	3.543	1.203	8.679	1	.003	1.186	5.900
	No	0[a]	.	.	0	.	.	.
	**B2B2C mode**							
	Yes	-.568	1.148	.245	1	.621	-2.818	1.683
	No	0[a]	.	.	0	.	.	.
	**Professional Management Department**							
	Has been established	.893	1.119	.637	1	.425	-1.300	3.087
	Being established	-2.303	1.473	2.445	1	.118	-5.190	.584
	Has not been established	0[a]	.	.	0	.	.	.
	**Management mode**							
	Self-management mode	5.090	3.674	1.919	1	.166	-2.111	12.292
	Partial entrustment mode	5.232	3.658	2.047	1	.153	-1.936	12.401
	Complete entrustment mode	.495	3.656	.018	1	.892	-6.670	7.661
	Other	0[a]	.	.	0	.	.	.
	**Investment amount(RMB)**							
	>5 million	-1.258	1.567	.645	1	.422	-4.330	1.813
	1~5 million	.009	1.040	.000	1	.993	-2.030	2.047
	500,000~1 million	-3.258	1.251	6.787	1	.009	-5.709	-.807
	100,000~500,000	-.609	.847	.516	1	.473	-2.269	1.052
	less than 100,000	0[a]	.	.	0	.	.	.
	**Government financial support**							
	Yes	-.700	.920	.580	1	.446	-2.502	1.102
	No	0[a]	.	.	0	.	.	.
	**Hospital self-raising**							
	Yes	-1.470	1.173	1.572	1	.210	-3.769	.828
	No	0[a]	.	.	0	.	.	.
	**Research funding**							
	Yes	3.722	1.508	6.094	1	.014	.767	6.677
	No	0[a]	.	.	0	.	.	.
	**Corporate sponsorship**							
	Yes	-.181	1.338	.018	1	.893	-2.802	2.441

No	0[a]	.	.	0	.	.	.
**Network Types**							
VPN	4.549	2.019	5.078	1	.024	.592	8.506
Public Internet	4.754	1.997	5.667	1	.017	.840	8.668
3G/4G	0[a]	.	.	0	.	.	.
**Data Storage**							
Independent storage	1.411	1.219	1.339	1	.247	-.979	3.801
Sharing with other departments	-.164	1.289	.016	1	.899	-2.691	2.363
Sharing with other hospitals	3.658	1.787	4.189	1	.041	.155	7.160
No storage	-2.432	1.319	3.397	1	.065	-5.018	.154
Other	0[a]	.	.	0	.	.	.
**Expertise level**							
Chief physician and above	5.292	2.370	4.986	1	.026	.647	9.938
Associate chief physician and above	1.011	.821	1.514	1	.219	-.599	2.621
Attending physician and above	0[a]	.	.	0	.	.	.
**Duration of case**							
>60 min	-11.099	3.499	10.062	1	.002	-17.958	-4.241
40-60 min	-5.498	2.961	3.447	1	.063	-11.302	.306
30-40 min	-7.832	3.047	6.608	1	.010	-13.804	-1.861
20-30 min	-6.077	2.892	4.415	1	.036	-11.744	-.409
10-20 min	-3.370	2.658	1.607	1	.205	-8.580	1.840
≤10min	0[a]	.	.	0	.	.	.
**Charge for telemedicine**							
Yes	4.257	1.023	17.304	1	.000	2.251	6.263
No	0[a]	.	.	0	.	.	.
**Waiting time**							
≤12h	-.315	2.096	.023	1	.880	-4.423	3.792
12-24h	1.035	2.133	.236	1	.627	-3.146	5.216
24-48h	3.537	2.063	2.940	1	.086	-.506	7.581
48-72h	0[a]	.	.	0	.	.	.

Link function: Logit.

a. Because this parameter is redundant, it is set to zero.
