# Supplementary material for: Implementation and Application of Telemedicine in China: Cross-Sectional Study
Source: JMIR Mhealth Uhealth. 2020 Oct 23;8(10):e18426. doi: 10.2196/18426 (PMC7647817; doi:10.2196/18426)
Supplement: Multimedia Appendix 3 [file mhealth_v8i10e18426_app3.pdf]

**Multimedia Appendix 2. Estimation results of ordinal regression analyzing the influencing factors of remote education effect**

| | | Coefficients | S.E. | Wald | df | P | 95% Confidence Interval | |
|---|---|---|---|---|---|---|---|---|
| | | | | | | | Lower | Upper |
| Threshold | No improvement | 8.975 | 11.839 | .575 | 1 | .448 | -14.228 | 32.178 |
| | Certain promotion | 19.317 | 12.387 | 2.432 | 1 | .119 | -4.961 | 43.596 |
| location | Computer science and Communication Professionals(number) | .002 | .325 | .000 | 1 | .995 | -.635 | .639 |
| | Medical professionals(number) | -.040 | .061 | .428 | 1 | .513 | -.159 | .079 |
| | Management professionals (number) | .143 | .196 | .532 | 1 | .466 | -.241 | .527 |
| | **Region** | | | | | | | |
| | East | -1.044 | 1.099 | .902 | 1 | .342 | -3.199 | 1.110 |
| | West | -1.657 | 1.162 | 2.033 | 1 | .154 | -3.933 | .620 |
| | Center | 0[a] | . | . | 0 | . | . | . |
| | **B2B mode** | | | | | | | |
| | Yes | 5.695 | 4.677 | 1.483 | 1 | .223 | -3.471 | 14.862 |
| | No | 0[a] | . | . | 0 | . | . | . |
| | **DTC mode** | | | | | | | |
| | Yes | 4.011 | 1.587 | 6.386 | 1 | .012 | .900 | 7.122 |
| | No | 0[a] | . | . | 0 | . | . | . |
| | **B2B2C mode** | | | | | | | |
| | Yes | -2.572 | 1.473 | 3.051 | 1 | .081 | -5.459 | .314 |
| | No | 0[a] | . | . | 0 | . | . | . |
| | **Professional Management Department** | | | | | | | |
| | Established | 1.450 | 1.265 | 1.313 | 1 | .252 | -1.030 | 3.929 |
| | Being established | 3.672 | 1.776 | 4.278 | 1 | .039 | .192 | 7.152 |
| | Not established | 0[a] | . | . | 0 | . | . | . |
| | **Investment amount(RMB)** | | | | | | | |
| | >5 million | .597 | 2.261 | .070 | 1 | .792 | -3.835 | 5.029 |
| | 1~5 million | .190 | 1.480 | .017 | 1 | .898 | -2.710 | 3.091 |
| | 500,000~1 million | -2.216 | 1.822 | 1.479 | 1 | .224 | -5.789 | 1.356 |
| | 100,000~500,000 | .385 | 1.122 | .118 | 1 | .732 | -1.815 | 2.585 |
| | less than 100,000 | 0[a] | . | . | 0 | . | . | . |
| | **Government financial support** | | | | | | | |
| | Yes | -2.223 | 1.222 | 3.308 | 1 | .069 | -4.619 | .173 |
| | No | 0[a] | . | . | 0 | . | . | . |
| | **Hospital self-raising** | | | | | | | |
| | Yes | -2.180 | 1.530 | 2.032 | 1 | .154 | -5.178 | .817 |
| | No | 0[a] | . | . | 0 | . | . | . |
| | **Research funding** | | | | | | | |
| | Yes | 4.691 | 1.825 | 6.605 | 1 | .010 | 1.114 | 8.269 |
| | No | 0[a] | . | . | 0 | . | . | . |
| | **Corporate sponsorship** | | | | | | | |
| | Yes | -1.769 | 1.649 | 1.150 | 1 | .283 | -5.002 | 1.464 |
| | No | 0[a] | . | . | 0 | . | . | . |
| | **Network Types** | | | | | | | |
| | VPN | 4.565 | 4.738 | .928 | 1 | .335 | -4.722 | 13.852 |
| | Public Internet | 3.291 | 4.704 | .489 | 1 | .484 | -5.929 | 12.511 |
| | 3G/4G | 0[a] | . | . | 0 | . | . | . |

| | | | | | | | |
|---|---|---|---|---|---|---|---|
| **Data Storage** | | | | | | | |
| Independent storage | -1.952 | 1.622 | 1.448 | 1 | .229 | -5.131 | 1.227 |
| Sharing with other departments | .823 | 1.680 | .240 | 1 | .624 | -2.470 | 4.116 |
| Sharing with other hospitals | .189 | 2.572 | .005 | 1 | .941 | -4.853 | 5.231 |
| No storage | -.756 | 1.739 | .189 | 1 | .664 | -4.164 | 2.652 |
| Other | 0[a] | . | . | 0 | . | . | . |
| **Management mode** | | | | | | | |
| Self-management mode | 7.551 | 9.873 | .585 | 1 | .444 | -11.800 | 26.902 |
| Partial entrustment mode | 9.674 | 9.964 | .943 | 1 | .332 | -9.854 | 29.203 |
| Complete entrustment mode | 2.866 | 9.886 | .084 | 1 | .772 | -16.509 | 22.242 |
| Other | 0[a] | . | . | 0 | . | . | . |
| **Frequency of distance education** | | | | | | | |
| 15 instances and above per month | 5.953 | 2.113 | 7.935 | 1 | .005 | 1.811 | 10.096 |
| 11-14 instances per month | -3.640 | 6.506 | .313 | 1 | .576 | -16.391 | 9.111 |
| 7-10 instances per month | 2.986 | 1.689 | 3.125 | 1 | .077 | -.324 | 6.296 |
| 4-6 instances per month | .138 | 1.109 | .016 | 1 | .901 | -2.036 | 2.312 |
| 0-3 times per month | 0[a] | . | . | 0 | . | . | . |
| **Charge for distance education** | | | | | | | |
| Yes | 4.304 | 1.525 | 7.967 | 1 | .005 | 1.315 | 7.293 |
| No | 0[a] | . | . | 0 | . | . | . |
| **Expertise level** | | | | | | | |
| Chief physician and above | -2.059 | 2.266 | .825 | 1 | .364 | -6.500 | 2.383 |
| Associate chief physician and above | -.422 | 1.090 | .150 | 1 | .699 | -2.558 | 1.714 |
| Attending physician and above | 0[a] | . | . | 0 | . | . | . |
| **Construction of distance education system** | | | | | | | |
| Yes | 1.874 | 1.166 | 2.582 | 1 | .108 | -.412 | 4.160 |
| No | 0[a] | . | . | 0 | . | . | . |

Link function: Logit.

a. Because this parameter is redundant, it is set to zero.
