# Supplementary material for: Implementation and Application of Telemedicine in China: Cross-Sectional Study
Source: JMIR Mhealth Uhealth. 2020 Oct 23;8(10):e18426. doi: 10.2196/18426 (PMC7647817; doi:10.2196/18426)
Supplement: Multimedia Appendix 4 [file mhealth_v8i10e18426_app4.pdf]

Multimedia Appendix 4. Checklist for reporting results of internet E-Surveys (CHERRIES)

Item category	*Checklist item*	*This study*
**Design**	Describe survey design	The purpose of the study was to investigate the construction and application of telemedicine in tertiary hospitals, and the participants were tertiary hospitals from 29 provinces, autonomous regions and municipalities in China. The survey was conducted through the Telemedicine Informationization Professional Committee of China (TICP). We firstly used the method of probability proportionate to size sampling (PPS) to calculate the sample size, and allocated sample sizes for different regions in proportion, then we obtained further research subjects through a snowball sampling method.
**IRB approval and informed consent process**	IRB approval	This study did not involve ethical issues.
	Informed consent	Informed consent was provided at the beginning of e-survey.
	Data protection	This study does not involve personal or family privacy. In order to ensure data security, we stored the research data on a local server and analyzed the data anonymously. The data will be used only for scientific research and will be kept strictly confidential.
**Development and pre-testing**	Development and testing	The preliminary questionnaire was developed based on the literature review and expert consultation, combined with telemedicine practice. 10 experts with more than five years experience in telemedicine were consulted about the questionnaire design. Further, before the formal large-scale investigation, a pre-survey was conducted in 18 tertiary hospitals in Henan province to offer their opinions about the items, permitting modification and improvement of the questionnaire.
**Recruitment process and description of the sample having access to the questionnaire**	Open survey versus closed survey	An open survey for each tertiary hospitals in China.
	Contact mode	Contact with participants online through email and WeChat.
	Advertising the survey	We issued a survey notice to each province through the Telemedicine Informationization Professional Committee of China (TICP), and determined the persons responsible for the survey in different provinces. The responsible person organized the survey in the tertiary hospitals in each region and used snowball techniques to promote the survey.

**Survey administration**	Web/E-mail	The survey was posted on a Web site.
	Context	A website dedicated to the questionnaire survey was designed, and all telemedicine staff in tertiary hospitals had access to the website. They completed the survey in their preferred location and at their preferred time.
	Mandatory/voluntary	Voluntary survey.
	Incentives	No incentives were provided for the participants.
	Time/date	October 2018.
	Randomization of items or questionnaires	No randomization.
	Adaptive questioning	N.A.
	Number of items	33 items in total.
	Number of screens (pages)	There are 11 items in per webpage and totally 3 webpages per survey.
	Completeness check	Completeness checks were conducted automatically before submitting the questionnaire. If the questionnaire was not completed, the website will give a reminder when submitting the questionnaire.
	Review step	Respondents were able to review and change their answers if they wished.
**Response rates**	Unique site visitor	Unique site visitors were determined by hospital name.
	View rate (ratio of unique survey visitors/unique site visitors)	Not available.
	Participation rate (ratio of unique visitors who agreed to participate/unique first survey page visitors)	Not available.
	Completion rate (ratio of users who finished the survey/users who agreed to participate)	161 out of 185 questionnaires were valid with a complete rate of 87.03%.
**Preventing multiple entries from the same individual**	Cookies used	No cookies were used.
	IP check	No IP check. We removed the duplicate questionnaires with the same hospital name.
	Log file analysis	The log file was not analyzed.
	Registration	N.A.
**Analysis**	Handling of incomplete questionnaires	Only completed questionnaires were analyzed.
	Questionnaires submitted with an atypical timestamp	No questionnaires were submitted with atypical timestamps.

Statistical correction	If the values of some items were invalid or missing, they were processed as missing values in the analysis of corresponding content.
